# Mortality after Parental Death in Childhood: A Nationwide Cohort Study from Three Nordic Countries

**DOI:** 10.1371/journal.pmed.1001679

**Published:** 2014-07-22

**Authors:** Jiong Li, Mogens Vestergaard, Sven Cnattingius, Mika Gissler, Bodil Hammer Bech, Carsten Obel, Jørn Olsen

**Affiliations:** 1 Section for Epidemiology, Department of Public Health, Aarhus University, Aarhus, Denmark; 2 Section for General Practice, Department of Public Health, Aarhus University, Aarhus, Denmark; 3 Research Unit for General Practice, Department of Public Health, Aarhus University, Aarhus, Denmark; 4 Clinical Epidemiology Unit, Department of Medicine, Karolinska Institute, Stockholm, Sweden; 5 National Institute for Health and Welfare (THL), Helsinki, Finland; 6 Research Programme for Mental Child Health, Department of Public Health, Aarhus University, Aarhus, Denmark; 7 Department of Epidemiology, School of Public Health, University of California, Los Angeles, California, United States of America; Umeå Centre for Global Health Research, Umeå University, Sweden

## Abstract

**Background:**

Bereavement by spousal death and child death in adulthood has been shown to lead to an increased risk of mortality. Maternal death in infancy or parental death in early childhood may have an impact on mortality but evidence has been limited to short-term or selected causes of death. Little is known about long-term or cause-specific mortality after parental death in childhood.

**Methods and Findings:**

This cohort study included all persons born in Denmark from 1968 to 2008 (*n* = 2,789,807) and in Sweden from 1973 to 2006 (*n* = 3,380,301), and a random sample of 89.3% of all born in Finland from 1987 to 2007 (*n* = 1,131,905). A total of 189,094 persons were included in the exposed cohort when they lost a parent before 18 years old. Log-linear Poisson regression was used to estimate mortality rate ratio (MRR). Parental death was associated with a 50% increased all-cause mortality (MRR = 1.50, 95% CI 1.43–1.58). The risks were increased for most specific cause groups and the highest MRRs were observed when the cause of child death and the cause of parental death were in the same category. Parental unnatural death was associated with a higher mortality risk (MRR = 1.84, 95% CI 1.71–2.00) than parental natural death (MRR = 1.33, 95% CI 1.24–1.41). The magnitude of the associations varied according to type of death and age at bereavement over different follow-up periods. The main limitation of the study is the lack of data on post-bereavement information on the quality of the parent-child relationship, lifestyles, and common physical environment.

**Conclusions:**

Parental death in childhood or adolescence is associated with increased all-cause mortality into early adulthood. Since an increased mortality reflects both genetic susceptibility and long-term impacts of parental death on health and social well-being, our findings have implications in clinical responses and public health strategies.

*Please see later in the article for the Editors' Summary*

## Abstract 2

Jiong Li and colleagues examine mortality rates in children who lost a parent before 18 years old compared with those who did not using population-based data from Denmark, Sweden, and Finland.

*Please see later in the article for the Editors' Summary*

## Editors' Summary

**Background:**

When someone close dies, it is normal to grieve, to mourn the loss of that individual. Initially, people who have lost a loved one often feel numb and disorientated and find it hard to grasp what has happened. Later, people may feel angry or guilty, and may be overwhelmed by feelings of sadness and despair. They may become depressed or anxious and may even feel suicidal. People who are grieving can also have physical reactions to their loss such as sleep problems, changes in appetite, and illness. How long bereavement—the period of grief and mourning after a death—lasts and how badly it affects an individual depends on the relationship between the individual and the deceased person, on whether the death was expected, and on how much support the mourner receives from relatives, friends, and professionals.

**Why Was This Study Done?:**

The loss of a life-partner or of a child is associated with an increased risk of death (mortality), and there is also some evidence that the death of a parent during childhood leads to an increased mortality risk in the short term. However, little is known about the long-term impact on mortality of early parental loss or whether the impact varies with the type of death—a natural death from illness or an unnatural death from external causes such as an accident—or with the specific cause of death. A better understanding of the impact of early bereavement on mortality is needed to ensure that bereaved children receive appropriate health and social support after a parent's death. Here, the researchers undertake a nationwide cohort study in three Nordic countries to investigate long-term and cause-specific mortality after parental death in childhood. A cohort study compares the occurrence of an event (here, death) in a group of individuals who have been exposed to a particular variable (here, early parental loss) with the occurrence of the same event in an unexposed cohort.

**What Did the Researchers Do and Find?:**

The researchers obtained data on everyone born in Denmark from 1968 to 2008 and in Sweden from 1973 to 2006, and on most people born in Finland from 1987 to 2007 (more than 7 million individuals in total) from national registries. They identified 189,094 individuals who had lost a parent between the age of 6 months and 18 years. They then estimated the mortality rate ratio (MRR) associated with parental death during childhood or adolescence by comparing the number of deaths in this exposed cohort (after excluding children who died on the same day as a parent or shortly after from the same cause) and in the unexposed cohort. Compared with the unexposed cohort, the exposed cohort had 50% higher all-cause mortality (MRR = 1.50). The risk of mortality in the exposed cohort was increased for most major categories of cause of death but the highest MRRs were seen when the cause of death in children, adolescents, and young adults during follow-up and the cause of parental death were in the same category. Notably, parental unnatural death was associated with a higher mortality risk (MRR = 1.84) than parental natural death (MRR = 1.33). Finally, the exposed cohort had increased all-cause MRRs well into early adulthood irrespective of child age at parental death, and the magnitude of MRRs differed by child age at parental death and by type of death.

**What Do These Findings Mean?:**

These findings show that in three high-income Nordic countries parental death during childhood and adolescence is associated with an increased risk of all-cause mortality into early adulthood, irrespective of sex and age at bereavement and after accounting for baseline characteristics such as socioeconomic status. Part of this association may be due to “confounding” factors—the people who lost a parent during childhood may have shared other unknown characteristics that increased their risk of death. Because the study was undertaken in high-income countries, these findings are unlikely to be the result of a lack of material or health care needs. Rather, the increased mortality among the exposed group reflects both genetic susceptibility and the long-term impacts of parental death on health and social well-being. Given that increased mortality probably only represents the tip of the iceberg of the adverse effects of early bereavement, these findings highlight the need to provide long-term health and social support to bereaved children.

**Additional Information:**

Please access these websites via the online version of this summary at http://dx.doi.org/10.1371/journal.pmed.1001679.

- The UK National Health Service Choices website provides information about bereavement, including personal stories; it also provides information about children and bereavement and about young people and bereavement, including links to not-for-profit organizations that support children through bereavement
- The US National Cancer Institute has detailed information about dealing with bereavement for the public and for health professionals that includes a section on children and grief (in English and Spanish)
- The US National Alliance for Grieving Children promotes awareness of the needs of children and teens grieving a death and provides education and resources for anyone who wants to support them
- MedlinePlus provides links to other resources about bereavement (in English and Spanish)

## Introduction

Bereavement by the death of a close relative is a major life event [1]. Spousal bereavement [2],[3] or child death in adult populations [4],[5] is often followed by an increased mortality risk. Maternal death in infancy or parental death in early childhood may lead to an increased mortality risk; however, scientific evidence is limited to short-term observations, historical data, or often studies in low- and mid-income settings [6]–[10]. In high-income countries, 3%–4% of children experience the death of a parent before they reach 18 years of age [11]. Such an early life adversity can have substantial impacts on future health in many ways [12],[13], but little is known about how it affect immediate or long-term mortality [3]. Further, the nature of the associations may vary with cause of death and type of death, but we are unaware of any large-scale studies on this, except a few indicating an excessive suicide risk following parental death [14],[15].

We hypothesized that parental death in early life has both short- and long-term impacts on health and social well-being, leading to an increased mortality risk accordingly over time. In addition to the genetic disposition of both physical [16],[17] and psychiatric diseases [14],[15],[18], psycho-social consequences following parental death [19] may play a significant role [12],[13]. Using combined nationwide data from three high-income countries (Denmark, Finland, and Sweden), we examined the association between parental death in children and adolescents and subsequent all-cause mortality risk until early or mid-adulthood, as well as cause-specific mortality. We further postulated that the magnitude of the associations differs according to sex of deceased parent, type and cause of parental death, child sex, and child age at parental death [3],[6],[8]–[10],[15],[20]. The unique data from national registers provided further opportunities to take the effects of socioeconomic inequality [21],[22] and other important factors into account [23]–[25].

## Methods

### Ethics Statement

The study was approved in Denmark by the Data Protection Agency and the Research Ethics Committee of the Central Region; in Sweden by the Research Ethics Committee (EPN) at the Karolinska Institute; and in Finland by Statistics Finland and the National Institute for Health and Welfare (THL). The study was based on encrypted data, on which the ethics committees do not require informed consent.

### Study Population

We established a population-based cohort study by combining nationwide data from three Nordic countries: Denmark, Sweden, and Finland [26]. In each country, the unique personal identification number allows accurate individual linkage of personal data from different national registers [27]. We included all children born in Denmark from 1968 to 2008 (*n* = 2,789,807) and all children born in Sweden from 1973 to 2006 (*n* = 3,380,301). A random sample of 89.3% of all children born in Finland from 1987 to 2007 (*n* = 1,131,905) were included, because Finland's authorities did not allow data to be recruited from 100% of the targeted population. As mortality during the first six months of life is mostly due to perinatal and congenital conditions [28], we started follow-up at six months of age. The exposure was defined as the death of a parent in the period from six months after birth to the day when a person turned 18 years of age [29]. Follow-up ended when the participants died, emigrated, or at the end of the study period (31 December 2009 in Denmark, 31 December 2008 in Sweden, and 31 December 2010 in Finland), whichever came first.

We excluded 711 children who died the same day as their parents (of whom 626 [87%] died from motor vehicle accidents, drowning, or other violent causes). We also excluded 94 children who died of the same or a related cause as their parents (defined by the same first two digits in the *International Statistical Classification of Diseases and Related Health Problems* [ICD] codes) within 30 days after having been exposed to parental death (of whom 76 [86%] died from motor vehicle accidents, drowning, or violent causes).

### Outcomes

The main outcomes of interest were all-cause mortality, cause-specific mortality, and type of death (natural death from diseases and medical conditions, unnatural death from external causes). We obtained information on the cause of death from the Cause of Death Register in each country. In Denmark, the eighth version (ICD-8) was used to categorize cause of death between 1978 and 1993 and the tenth version (ICD-10) between 1994 and 2007. In Sweden, the ICD-8 was used between 1973 and 1986, the ICD-9 between 1987 and 1996, and the ICD-10 between 1997 and 2008. In Finland, the ICD-9 was used between 1987 and 1995 and the ICD-10 between 1996 and 2010 [26]. Type of death was separated into two categories (natural death from diseases and medical conditions [ICD-8 codes 000-799, ICD-9 codes 000-799, and ICD-10 codes A00-R99], and unnatural death from external causes [ICD-8 codes E800-E999, ICD-9 codes E800-E999, and ICD-10 codes V01-Y98]). Cause-specific mortality was defined according to the main chapters in the ICD system as the following ten groups: infections and parasitic diseases (ICD 8 and ICD 9 codes 000–1399, IC 10 codes A00–B999); neoplasms (ICD 8 and ICD 9 codes 140–2389, ICD 10 codes C00–C999); endocrine, nutritional, and metabolic diseases (ICD 8 and ICD 9 codes 240–2799, ICD 10 codes E00–E909); mental and behavioural disorders (ICD 8 and ICD 9 codes 290–3159, ICD 10 codes F00–F999); diseases of the nervous system and the sense organs (ICD 8 and ICD9 codes 320–389, ICD 10 codes G00–H95); diseases of the circulatory system (ICD 8 and ICD9 codes 410–414, 420–423, 425–429, ICD 10 codes I20–I25, I30–I33, I39–I52); diseases of the respiratory system (ICD 8 and ICD9 codes 460–519, ICD 10 codes J00–J99); diseases of the digestive system (ICD 8 and ICD9 codes 520–579, 4442, ICD 10 codes K00–K93); transport accidents (ICD 8 and ICD9 codes 810–823, ICD 10 codes V01–V89); and suicide and intentional self-harm (ICD 8 and ICD9 codes 950–959, ICD 10 codes X60–X84).

### Information on Potential Confounders and Risk Factors

Information on child sex, birth characteristics (birth weight, gestational age, and Apgar score at five minutes, etc.), maternal age, and parity was retrieved from the national Medical Birth Registers (MBRs) [26]. These registers hold recorded information from antenatal, obstetric, and neonatal medical records on practically all deliveries. Information on socioeconomic status (maternal education and social status) was obtained from the Danish Integrated Database for Labor Market Research (IDA) in Denmark, the Swedish Register of Education in Sweden, and in Finland from Statistics Finland and THL [26].

### Statistical Analysis

Data were analyzed using log-linear Poisson regression models (SAS Genmod procedure, version 9.2) as an approximation of the Cox regression, as the latter would often be too computationally intensive for a dataset of this size with time-dependent variables [30].

The exposure was treated as a time-varying variable, i.e., all persons were allocated to the unexposed cohort at the beginning of the follow-up (0.5 years of age). Those who lost a parent before they reached 18 years of age would be moved to the exposed cohort from the day when the parent died. All children who did not lose a parent before they reached 18 years of age remained in the unexposed cohort. Follow-up time was counted by days as offset variable in the model. Child age was a categorical variable defined by the age in each calendar year. In some analyses, the length of follow-up time was categorized into six periods (0–2 years, 3–6 years, 7–10 years, 11–14 years, 15–18 years, ≥19 years).

Mortality rate ratios (MRRs) for the exposed and the unexposed were estimated according to all-cause mortality, type of child death (natural death, unnatural death), and cause-specific mortality. When using natural death as response (outcome), unnatural death is a competing event and treated as a censored case. We did the same for cause-specific mortality, performing ten separate analyses for the ten above-mentioned main cause groups. For each of these ten analyses, we separated the exposure into two sub-categories: the first sub-category of “same cause” referred to a cause of parental death that belonged to the same specific-cause group of child mortality and others were grouped into the second exposed sub-category of “not same cause.” For example, if a specific-cause group of child mortality (outcome) was “Infections & parasitic diseases,” the first exposure sub-category was parental death due to “Infections & parasitic diseases,” the second exposure sub-category was parental death due to other causes than “Infections & parasitic diseases.” This approach would to some extent help us to evaluate the role of genetic disposition for cause-specific mortality.

In additional analyses, we examined the MRRs according to specific cause groups of parental death. We further analyzed data according to sub-categories of exposure: child age at parental death (6 months–4 years, 5–10 years, 11–14 years, and 15–18 years), sex of the deceased parent (father, mother), type of parental death (natural death, unnatural death).

We also performed subgroup analyses based on specific characteristics of the study population, such as country, sex of child, child birth characteristics, and maternal socioeconomic status.

The following potential confounders were included in the model: country (Denmark, Sweden, and Finland), sex (male, female), and birth characteristics including birth weight (<2,500 g, 2,500–3,249 g, 3,250–3,999 g, ≥4,000 g), preterm birth (gestational age: <37 weeks, ≥37 weeks), and Apgar score at five minutes (1–8, 9–10). We also included maternal socio-demographic characteristics at childbirth, such as age (≤26, 27–30, ≥31 years), parity (1st, 2nd, 3rd, or higher), education (low [≤9 years], middle [10–14 years], and high [≥15 years] [available for Swedish data from 1990, 1995, 2000, and 2005; for annual Danish data from 1980 to 2007; and for annual Finnish data from 1987 to 2007]), social status (1 = not in labor market; 2 = unskilled worker, 3 = skilled worker and white collar; 4 = high status, such as medium to big business owners, top administrative officials; 9 = missing values; data were available for the periods 1980–2008 in Denmark, 1980, 1985, 1990 in Sweden, and 1990–2007 in Finland), and data on smoking in early pregnancy ([yes, no] was available for the periods 1983–2006 in Sweden, 1991–2007 in Denmark, and 1987–2007 in Finland).

## Results

### Study Population Characteristics, Overall Estimates

Out of 7,302,013 individuals included in this study, 189,094 (2.6%) lost a parent in the period from 6 months of age to 18 years of age. The exposed and the unexposed cohorts were comparable in terms of most baseline characteristics at birth, except that more mothers of exposed children tended to have a short-term education and high parity, and more mothers of exposed children smoked during pregnancy, and fewer had the highest social status (Table 1).

**Table 1:** Characteristics of the exposed and unexposed cohort.

Variables	Number Exposed Cohort^a^ (%)	Number Unexposed Cohort^a^ (%)
**Country**		
Denmark	89,905 (48)	2,699,902 (37)
Sweden	83,639 (44)	3,296,662 (46)
Finland	15,550 (8)	1,116,355 (16)
**Sex**		
Boy	96,940 (51)	3,647,618 (51)
Girl	92,154 (49)	3,465,301 (49)
**Preterm birth** b		
Yes	11,798 (7)	361,408 (5)
No	152,401 (89)	6,155,416 (91)
Unknown	6,103 (4)	239,344 (4)
**Singleton** b		
Yes	16,359 (96)	6,442,449 (95)
No	4,140 (2)	175,620 (3)
Unknown	2,571 (2)	158,099 (2)
**Birth weight** b		
<2,500 g	9,360 (6)	260,756 (4)
2,500–3,249 g	43,509 (29)	1,545,832 (24)
3,250–3,999 g	72,055(48)	3,265,065 (51)
≥4,000 g	22,622 (15)	1,119,185 (18)
Unknown	2519 (2)	160,136 (3)
**Maternal age at child birth (y)**		
≤26	67,351 (34)	2,699,329 (38)
27–30	46,748 (25)	1,989,534 (28)
≥31	74,881 (40)	2,412,940 (32)
Unknown	114 (<1)	11,116 (<1)
**Parity** b		
1	64,206 (34)	2,554,719 (36)
2	53,611 (28)	1,483,838 (21)
≥3	67,801 (36)	3,040,273 (43)
Unknown	3476 (2)	34,089 (<1)
**Apgar score at 5 minutes** b		
1–8	5,618 (3)	219,710 (3)
9–10	124,140 (70)	4,999,175 (78)
Unknown	48,412 (27)	1,198,523 (19)
**Maternal education at child birth** c		
Low, ≤9 years	42,179 (32)	1,099,544 (21)
Middle, 10–14 years	54,924 (42)	2,584,874 (50)
High, ≥15 years	18,035 (14)	1,057,300 (20)
Unknown	16,493 (13)	432,255 (8)
**Maternal social status at child birth** c		
Not in labor market	24,469(17)	956,976 (16)
Unskilled workers	30,168 (21)	1,191,848 (20)
Skilled workers/white collars	33,429 (24)	1,824,232 (30)
Top level status	17,725 (13)	1,040,598 (17)
Unknown	37,842 (25)	1,055,008 (17)
**Maternal Smoking during pregnancy**		
Yes	27,721 (34)	817,171 (18)
No	46,783 (57)	3,412,915 (75)
Unknown	7,206 (9)	336,262 (7)

a Shown are number of study participants.

b Birth weight available period: 1979–2008 in Denmark, 1973–2006 in Sweden, 1987–2007 in Finland; parity available period: 1968–2008 in Denmark, 1973–2006 in Sweden, 1987–2007 in Finland; Gestational age and singleton available period: 1973–2008 in Denmark, 1973–2006 in Sweden, 1987–2007 in Finland. Apgar score at 5 minutes: 1978–2008 in Denmark, 1973–2006 in Sweden, 1987–1989, 2003–2007 in Finland.

c Maternal education available period: 1980–2007 in Denmark, 1990, 1995, 2000, 2005 in Sweden, and 1987–2007 in Finland; Maternal smoking during pregnancy available period: 1991–2007 in Denmark, 1982–2006 in Sweden, 1987–2007 in Finland; Maternal social status available period: 1980–2008 in Denmark, 1980, 1985, 1990 in Sweden, 1990–2007 in Finland.

During the follow-up period, 39,683 individuals included in this study died. Compared with the unexposed cohort, the exposed cohort had a 50% higher all-cause mortality (MRR = 1.50, 95% CI 1.43–1.58) (Table 2). The results were similar when the analyses were stratified by the individual's sex or the deceased parent's sex (Table 3). The relative risk of natural death (from diseases or medical conditions) in the exposed cohort (MRR = 1.45, 95% CI 1.34–1.58) was slightly but not significantly lower than that of unnatural death (external causes) (MRR = 1.60, 95% CI 1.49–1.71). Compared to the unexposed cohort members, the exposed individuals whose parents died by unnatural death had an 84% increased risk of all-cause mortality risk (MRR = 1.84, 95% CI 1.71–2.00), which was higher than the 33% increased risk in those whose parents died by natural death (MRR = 1.33, 95% CI 1.24–1.41). This difference was mainly contributed by a high MRR (MRR = 2.15, 95% CI 1.94–2.38) when both the child and the parent died by unnatural death (Table 2). For child natural death, parental unnatural death and parental natural death were associated with similar MRRs (MRR = 1.45, 95% CI 1.30–1.60 versus MRR = 1.44, 95% CI 1.26–1.67, respectively) (Table 2).

**Table 2:** Mortality rate ratios after parental death in childhood, by type of death.

Outcome (Child Mortality)	Exposure (Type of Parental Death)	Cases/Person Years (Rate, 1/10^5^)	MRR (95% CI) Model 1^a^	MRR (95% CI) Model 2^b^
**All death**	**All parental death**	**1,695/2,679,043 (63.3)**	**1.58 (1.51–1.66)** *	**1.50 (1.43–1.58)** *
	Parental natural death	995/1,733,666 (57.4)	1.63 (1.53–1.74)*	1.33 (1.24–1.41)*
	Parental unnatural death	670/904,568 (74.1)	2.21 (2.04–2.38)*	1.84 (1.71–2.00)*
	**Unexposed**	37,988/129,341,810 (29.4)	1.0 (ref)	1.0 (ref)
**Natural death**	**All parental death**	**603/2,679,043 (22.5)**	**1.56 (1.43–1.69)** *	**1.45 (1.34–1.58)** *
	Parental natural death	394/1,733,371 (22.7)	1.48 (1.29–1.70)^8^	1.44 (1.26–1.67)*
	Parental unnatural death	197/904,329 (21.8)	1.53 (1.38–1.69)^8^	1.45 (1.30–1.60)*
	**Unexposed**	18,709 (14.5)	1.0 (ref)	1.0 (ref)
**Unnatural death**	**All parental death**	**902/2,679,043 (33.7)**	**1.65 (1.54–1.77)** *	**1.60 (1.49–1.71)** *
	Parental natural death	495/1,733,666 (28.5)	1.79 (1.63–1.95)*	1.32 (1.20–1.44)*
	Parental unnatural death	394/904,568 (43.6)	2.92 (2.63–3.22)*	2.15 (1.94–2.38)*
	**Unexposed**	15,890 (12.3)	1.0 (ref)	1.0 (ref)

a MRRs were adjusted for country, age, and sex.

b MRRs were adjusted for country, age, sex, calendar year period, birth outcomes (birth weight, the Apgar score at 5 minutes, preterm birth), and maternal variables (age, parity, education, and social status).

*p<0.05.

**Table 3:** Mortality rate ratios after parental death, according to sex of parent, sex of child.

Sex of Deceased Parent	Child Sex	Cases/Person Years (Rate, 1/10^5^)	MRR (95% CI) Model 1^a^	MRR (95% CI) Model 2^b^
**Death of any parent**	**Both gender**	**1,695/2,679,043 (63.3)**	**1.58 (1.51–1.66)** *	**1.50 (1.43–1.58)** *
	Boys only	1,203/1,379,764 (87.2)	1.60 (1.51–1.70)*	1.54 (1.45–1.64)*
	Girls only	492/1,299,276 (37.9)	1.52 (1.39–1.67)*	1.43 (1.30–1.56)*
**Death of a father**	**Both gender**	**1,146/1,877,223 (61.4)**	**1.54 (1.45–1.63)** *	**1.50 (1.41–1.59)** *
	Boys only	819/964,273 (84.9)	1.67 (1.51–1.85)*	1.58 (1.43–1.76)*
	Girls only	327/915,951 (35.7)	1.45 (1.30–1.62)*	1.37 (1.23–1.54)*
**Death of a mother**	**Both gender**	**549/808,120 (67.9)**	**1.67 (1.54–1.82)** *	**1.55 (1.43–1.70)** *
	Boys only	384/415,491 (92.4)	1.63 (1.48–1.80)*	1.55 (1.40–1.72)*
	Girls only	165/386,325 (42.7)	1.68 (1.44–1.97)*	1.51 (1.29–1.78)*

a MRRs were adjusted for country, age, and sex.

b MRRs were adjusted for country, age, sex, calendar year period, birth outcomes (birth weight, the Apgar score at 5 minutes, preterm birth), and maternal variables (age, parity, education, and social status).

*p<0.05.

### Subgroup Analyses on Specific Characteristics of the Study Population

Although the absolute mortality rate was twice as high among boys (87.2/10^5^ person-years) as among girls (37.9/10^5^ person-years), similar MRR estimates were observed in the analyses stratified by child sex (in boys MRR = 1.54, 95% CI 1.45–1.64, in girls MRR = 1.43, 95% CI 1.30–1.56) (Table 3). Analyses further performed by the deceased parent's sex yielded similar findings (Table 3). With only a few exceptions, subgroup analyses on specific baseline characteristics of the study population showed similarly elevated relative risk estimates (MRRs) across sub-categories (Table 4), although there were significant variations in absolute mortality rates between sub-categories of population characteristics. For example, compared to children born to mothers with a low social status (“unskilled workers” as the reference group), children of mothers with higher social status groups (“skilled workers/white collars” and “top level status”) had an 8% reduced risk (MRR  =  0.92, 95% CI 0.86–0.97) and a 13% reduced risk (MRR  =  0.87, 95% CI 0.84–0.89) of mortality, respectively; while those of mothers who were not in labor market had a 5% increased mortality risk (MRR = 1.05, 95% CI 1.01–1.09) (Table 5). But except those with top level status, we observed similar magnitudes of the association between parental death and mortality in other three sub-categories, 1.42 (1.22–1.65) for “not in labor market,” 1.50 (1.31–1.75) for “unskilled workers,” and 1.36 (1.16–1.61) for “skilled workers/white collars,” respectively (Table 4).

**Table 4:** Mortality rate ratios according to characteristics of study population.

Variables	Cases in the Exposed/Unexposed	Mortality Rate in the Exposed/Unexposed	MRR^a^
**Country**			
Denmark	1,053/19,210	73.4/35.7	1.53 (1.44–1.63)*
Sweden	582/15,629	51.6/25.8	1.47 (1.35–1.60)*
Finland	108/2,769	72.5/46.7	1.39 (1.14–1.70)*
**Preterm birth** b			
Yes	108/2,769	72.6/46.7	1.39 (1.14–1.70)*
No	1,078/27,662	54.9/26.2	1.53 (1.43–1.63)*
**Singleton** b			
Yes	1,197/30,456	56.4/27.4	1.51 (1.42–1.60)*
No	21/855	43.4/32.6	1.10 (0.70–1.63)
**Birth weight** b			
<2,500 g	65/2,416	59.7/57.8	1.04 (0.81–1.35)
2,500–3,249 g	273/7,310	51.8/28.2	1.47 (1.30–1.66)*
3,250–3,999 g	393/11,920	45.9/22.3	1.50 (1.35–1.66)*
≥4,000 g	144/3,941	55.7/22.3	1.71 (1.45–2.03)*
**Apgar score at 5 minutes** b			
1–8	57/2,823	64.8/47.2	1.09 (0.83–1.44)
9–10	722/2,817	48.6/24.2	1.48 (1.37–1.59)*
Unknown	28/19,795	44.5/16.3	2.13 (1.40–3.27)*
**Maternal age at child birth**			
≤26	766/19,340	73.4/34.0	1.56 (1.45–1.68)*
27–30	380/9,446	57.3/25.8	1.47 (1.34–1.60)*
≥31	573/9,092	56.4/25.1	1.43 (1.32–1.56)*
**Parity** b			
1	591/15,774	62.4/28.5	1.58 (1.45–1.74)*
2	539/13,350	60.5/28.5	1.50 (1.38–1.64)*
≥3	474/8,570	64.0/32.2	1.41 (1.29–1.55)*
**Maternal education at child birth** b			
≤9 years	269/6,899	53.3/32.9	1.35 (1.19–1.53)*
10–14 years	289/10,709	45.3/23.6	1.51 (1.34–1.70)*
≥15 years	63/3141	32.9/21.2	1.32 (1.04–1.72)*
**Maternal social status at child birth** b			
Not in labor market	191/5,338	55.7/31.1	1.42 (1.22–1.65)*
Unskilled workers	196/5,404	54.3/25.4	1.50 (1.31–1.75)*
Skilled workers/white collars	157/6,658	40.7/22.0	1.36 (1.16–1.61)*
Top level status	97/4,049	43.9/24.3	1.24 (1.01–1.53)*
**Maternal smoking during pregnancy** b			
Yes	108/3,062	46.3/25.0	1.47 (1.20–1.80)*
No	126/7,929	33.6/18.1	1.52 (1.26–1.82)*

a MRRs were adjusted for country, age, sex, calendar year period, birth outcomes (birth weight, the Apgar score at 5 minutes, preterm birth), and maternal variables (age, parity, education, and social status).

b Birth weight available period: 1979–2008 in Denmark, 1973–2006 in Sweden, 1987–2007 in Finland; parity available period: 1968–2008 in Denmark, 1973–2006 in Sweden, 1987–2007 in Finland; Gestational age and singleton available period: 1973–2008 in Denmark, 1973–2006 in Sweden, 1987–2007 in Finland. Apgar score at 5 minutes: 1978–2008 in Denmark, 1973–2006 in Sweden, 1987–1989, 2003–2007 in Finland; Maternal education available period: 1980–2007 in Denmark, 1990, 1995, 2000, 2005 in Sweden, and 1987–2007 in Finland; Maternal smoking during pregnancy available period: 1991–2007 in Denmark, 1982–2006 in Sweden, 1987–2007 in Finland; Maternal social status available period: 1980–2008 in Denmark, 1980, 1985, 1990 in Sweden, 1990–2007 in Finland.

*p<0.05.

**Table 5:** Mortality rate ratios among sub-categories of population baseline characteristics.

Variables	MRR^a^
**Country**	
Denmark	1.21 (1.17–1.26)*
Sweden	1.0 (ref)
Finland	1.06 (0.99–1.16)
**Sex**	
Boy	1.78 (1.74–1.81)*
Girl	1.0 (ref)
**Preterm birth** b	
Yes	1.12 (1.06–1.17)*
No	1.0 (ref)
**Singleton** b	
Yes	0.79 (0.73–0.85)*
No	1.0 (ref)
**Birth weight** b	
<2,500 g	2.14 (2.03–2.27)
2,500–3,249 g	1.22 (1.19–1.28)*
3,250–3,999 g	1.0 (ref)
≥4,000 g	0.97 (0.93–0.99)*
**Apgar score at 5 minutes** b	
1–8	1.99 (1.87–2.06)*
9–10	1.0 (ref)
**Maternal age at child birth**	
≤26	1.23 (1.20–1.25)*
27–30	1.02 (1.00–1.05)*
≥31	1.0 (ref)
**Parity** b	
1	1.0 (ref)
2	1.09 (1.06–1.11)*
≥3	1.31 (1.28–1.35)*
**Maternal education at child birth** b	
≤9 years	1.0 (ref)
10–14 years	0.82 (0.77–0.84)*
≥15 years	0.80 (0.76–0.85)*
**Maternal social status at child birth** b	
Not in labor market	1.05 (1.01–1.09)*
Unskilled workers	1.0 (ref)
Skilled workers/white collars	0.92 (0.86–0.97)*
Top level status	0.87 (0.84–0.89)*
**Maternal smoking during pregnancy** b	
Yes	1.21 (1.15–1.24)*
No	1.0 (ref)

a MRRs were adjusted for country, age, sex, calendar year period, birth outcomes (birth weight, the Apgar score at 5 minutes, preterm birth), and maternal variables (age, parity, education, and social status).

b Birth weight available period: 1979–2008 in Denmark, 1973–2006 in Sweden, 1987–2007 in Finland; parity available period: 1968–2008 in Denmark, 1973–2006 in Sweden, 1987–2007 in Finland; Gestational age and singleton available period: 1973–2008 in Denmark, 1973–2006 in Sweden, 1987–2007 in Finland. Apgar score at 5 minutes: 1978–2008 in Denmark, 1973–2006 in Sweden, 1987–1989, 2003–2007 in Finland; Maternal education available period: 1980–2007 in Denmark, 1990, 1995, 2000, 2005 in Sweden, and 1987–2007 in Finland; Maternal smoking during pregnancy available period: 1991–2007 in Denmark, 1982–2006 in Sweden, 1987–2007 in Finland; Maternal social status available period: 1980–2008 in Denmark, 1980, 1985, 1990 in Sweden, 1990–2007 in Finland.

*p<0.05.

### Child Cause-Specific Mortality

The exposed cohort had higher mortality risks from most major groups of cause of death than the unexposed cohort (Table 6), for example death from the nervous system (MRR = 1.71, 95% CI 1.36–2.17), the digestive system (MRR = 2.34, 95% CI 1.61–3.38), the circulatory system (MRR = 1.80, 95% CI 1.36–2.37), and suicide and intentional self-harm (MRR = 1.78, 95% CI 1.56–2.03). Highest MRR estimates were seen if children died from the same cause as the deceased parent (the first exposure sub-category: “same cause”), for example the 7-fold or 5-fold relative risks of mortality from nervous system diseases or digestive diseases, respectively. However, the first exposure sub-category (“same cause”) often accounted for only a minor proportion of the exposed group, and the second exposed category (“not same cause”) had a slightly attenuated but similar MRR estimates to the average ones for most specific cause groups.

**Table 6:** Cause-specific mortality rate ratios after parental death in childhood.

Outcome (Cause of Death in the Offspring)	Exposure (Cause of Parental Death)^a^	Number Deaths in the Exposed/the Unexposed	Rate in the Exposed/the Unexposed (1/10^5^)	MRR (95% CI)^b^
**Infections and parasitic diseases**	**All causes**	**21/999**	**0.74/0.77**	**1.40 (0.90–2.18)**
	Same cause	0/998	0/0.77	—
	Not same cause	21/998	0.78/0.77	1.40 (0.90–2.17)
**Diseases of the nervous system**	**All causes**	**76/2,166**	**2.81/1.66**	**1.71 (1.36–2.17)** *
	Same cause	4/2,166	10.5/1.66	7.26 (2.94–15.91)*
	Not same cause	72/2,166	2.49/1.66	1.64 (1.24–2.14)*
**Diseases of the respiratory system**	**All causes**	**20/956**	**0.71/0.74**	**1.02 (0.63–1.64)**
	Same cause	2/956	2.11/0.74	2.12 (0.53–8.51)
	Not same cause	18/956	0.69/0.74	2.10 (0.61–1.55)
**Diseases of the digestive system**	**All causes**	**32/442**	**1.20/0.34**	**2.34 (1.61–3.38)** *
	Same cause	4/442	2.13/0.34	4.65(1.73–12.47)*
	Not same cause	28/442	1.09/0.34	2.08 (1.42–3.05)*
**Diseases of the circulatory system**	**All causes**	**56/960**	**2.15/0.74**	**1.80 (1.36–2.37)** *
	Same cause	10/960	1.70/0.74	1.87 (1.00–3.51)*
	Not same cause	46/960	1.97/0.74	1.71 (1.27–2.31)*
**Neoplasms**	**All causes**	**172/5,312**	**6.35/4.11**	**1.32 (1.13–1.54)** *
	Same cause	67/5,312	8.31/4.11	1.71 (1.34–2.18)*
	Not same cause	105/5,312	5.60/4.11	1.16 (0.96–1.41)
**Endocrine, nutritional and metabolic diseases**	**All causes**	**33/1,085**	**1.41/0.83**	**1.26 (0.88–1.82)**
	Same cause	1/1,085	1.19/0.83	1.25 (0.18–8.89)
	Not same cause	32/1,085	1.22/0.83	1.26 (0.87–1.82)
**Mental and behavioral disorders**	**All causes**	**25/340**	**1.02/0.26**	**1.57 (1.03–2.40)** *
	Same cause	1/340	1.05/0.26	3.77 (1.56–9.08)*
	Not same cause	24/340	0.91/0.26	1.64 (1.28–2.08)*
**Transport accidents**	**All causes**	**294/6,647**	**11.14/5.12**	**1.21 (1.07–1.38)** *
	Same cause	21/6,647	7.77/5.12	1.21 (0.78–1.85)
	Not same cause	273/6,647	10.95/5.12	1.25 (1.10–1.41)*
**Suicide and intentional self-harm**	**All causes**	**273/3,536**	**10.19/2.72**	**1.78 (1.56–2.03)** *
	Same cause	64/3,536	10.03/2.72	2.78 (2.17–3.57)*
	Not same cause	209/3,536	9.23/2.72	1.57 (1.36–1.81)*

a Same cause: parental death cause is of same cause group as the child death cause; Not same cause: parental death cause is not of same cause group as the child death cause.

b MRRs were adjusted for country, age, sex, calendar year period, birth outcomes (birth weight, the Apgar score at 5 minutes, preterm birth), and maternal variables (age, parity, education, and social status).

*p<0.05.

### Child Mortality According to Specific Cause of Parental Death

**Figure 1:**
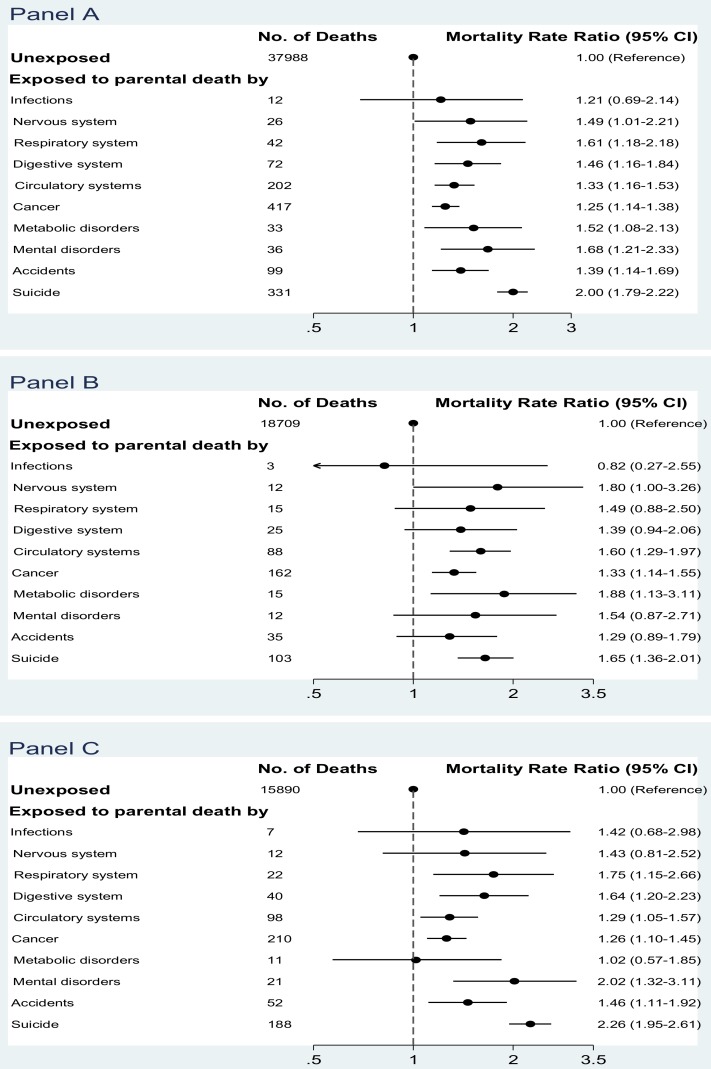
Mortality rate ratios after parental death in childhood, according to specific cause of parental death. (A) All-cause mortality in children; (B) mortality from natural death in children; (C) mortality from unnatural death in children (MRRs were adjusted for country, age, sex, calendar year period, birth outcomes [birth weight, the Apgar score at 5 minutes, preterm birth], and maternal variables [age, parity, education, and social status]).

All-cause mortality in the exposed cohort was increased for almost all specific causes of parental death, albeit the MRRs were not statistically significant for several causes like infections or diseases of the nervous system (Figure 1A). The highest all-cause MRR was seen for individuals who lost a parent due to suicide (MRR = 2.00, 95% CI 1.79–2.22) (Figure 1A). The magnitude of the associations varied according to the two types of child death (natural death in Figure 1B, unnatural death in Figure 1C). For example, parental suicide was associated with a 65% increased risk of child natural death (MRR = 1.65, 95% CI 1.36–2.01) (Figure 1B), and an even higher MRR of 2.26 (95% CI 1.95–2.61) of child unnatural death (Figure 1C). Parental suicide was also associated with an increased risk of child accidental death (MRR = 1.37, 95% CI 1.03–1.82) (Table 7).

**Table 7:** Child suicide and accident mortality rate ratios after parental death, according to parental death from suicide and death.

Child Mortality	Exposure	Cases	MRR (95% CI) Model 1^a^	MRR (95% CI) Model 2^b^
**Suicide**	Parental suicide	64	3.06 (2.39–3.93)*	2.87 (2.24–3.67)*
	Parental accidental death	14	1.66 (0.96–2.86)	1.53 (0.89–2.65)
**Accidents**	Parental suicide	49	1.43 (1.06–1.86)*	1.37 (1.03–1.82)*
	Parental accidental death	21	1.49 (0.97–2.31)	1.41 (0.92–2.17)

a MRRs were adjusted for country, age, and sex.

b MRRs were adjusted for country, age, sex, calendar year period, birth outcomes (birth weight, the Apgar score at 5 minutes, preterm birth), and maternal variables (age, parity, education, and social status).

*p<0.05.

### Child Mortality according to Child Age at Bereavement and Length of Follow-up Time

**Figure 2:**
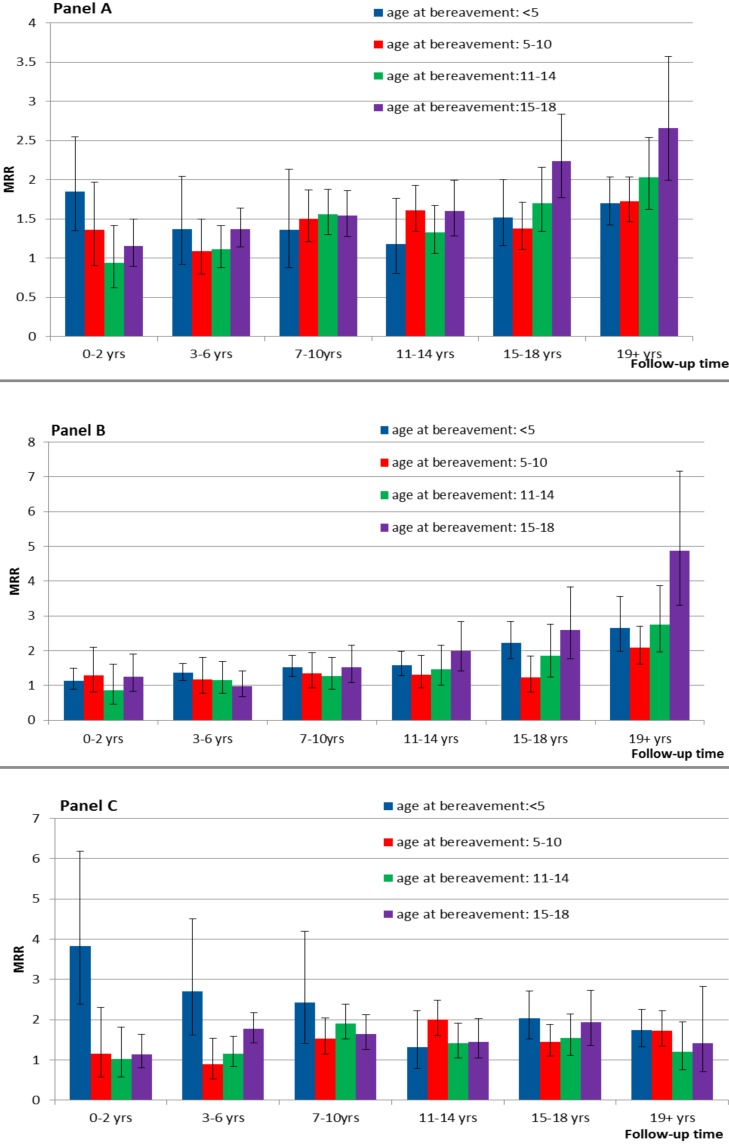
Mortality rate ratios after parental death in childhood by the length of follow-up time, according to child age at bereavement. (A) All-cause death MRRs over follow-up, by age at bereavement; (B) natural death MRRs over follow-up, by age at bereavement; (C) unnatural death MRRs over follow-up, by age at bereavement (MRRs were adjusted for country, age, sex, calendar year period, birth outcomes [birth weight, the Apgar score at 5 minutes, preterm birth], and maternal variables [age, parity, education, and social status]).

The exposed cohort had increased all-cause mortality MRRs well into early adulthood, irrespective of child age at parental death. The magnitude of MRRs differed by child age at parental death and type of death. For natural death, there was a tendency that MRRs increased over follow-up time, while those who were exposed at advanced child age groups tended to have higher MRRs in the later periods of follow-up. For unnatural death, those exposed before 5 years of age mostly had higher MRRs than others, especially at the beginning years of follow-up. Those exposed at age 15–18 also had relatively high MRRs throughout the follow-up periods (Figure 2).

## Discussion

In this large population-based cohort study, parental death in childhood and adolescence was associated with an increased risk of all-cause mortality that persisted into early adulthood, irrespective of sex and age at bereavement and after accounting for the effects of specific baseline characteristics like socioeconomic status and birth characteristics. The elevated risks were seen for almost all major cause of death groups and the highest risks were observed when children died from the same cause as the parent. Parental unnatural death was associated with a higher risk than parental natural death. Increased risks of child mortality were observed for almost all major cause groups of parental death.

The increased overall mortality following parental death is in line with the suggestions from historical populations and from low-income countries [6]–[10]. However, in these studies parents and children might often die from lack of material or health care needs [6]–[10]. In high-income countries, although many children may suffer significantly from parental death [19], very few of them seem to die because of a lack of basic needs. The increased relative risk was seen both in the short and long run, irrespective of socioeconomic inequalities in mortality [21],[22] or other variables, although there were variations in magnitude of associations within the baseline characteristics. Further, in contrast with the results from some studies in which only the death of a mother matters [7],[31], we also observed that the death of a father was associated with a similar long-term increased risk. Maternal care might be relatively more critical for child short-term survival [7],[31], but in a long-term perspective paternal care is also of importance, both directly in child care and in providing economic or emotional support [32]–[34].

How parental loss in childhood influences mortality risk from physical diseases is not well studied [19]. We are the first, to our knowledge, to show a long-term increased risk of death from multiple major disease groups following parental death in the early years of life. As observed, genetic disposition may play a significant role as family members have an increased risk of dying from certain diseases [16],[17]. However genetic susceptibility most likely explains only a minor part of the observed association. Again, an increased risk of natural death was seen even if the parent died from an unnatural cause. A growing body of evidence suggests that early life adversities negatively affect future health and social well-being, both by biological and psycho-social/behavioral mechanisms [12],[13]. First, parental death has been shown to have long-lasting biological effects, such as hypothalamic-pituitary-adrenal dysfunction [35],[36] and the metabolic syndrome later in life [37]. Recent research supports a molecular basis of the interaction between genetic susceptibility and early stress exposure, such as epigenetic mechanisms of a global effect on immune function [13],[38]. Another potential pathway could be the shared environmental settings, as reported in widowed populations [2],[3]. Furthermore, in this study setting, parental death may lead to social or economic disadvantages that can result in an increased mortality risk [21],[22]. Personal lifestyle changes following parental death, especially more risk behaviors, may contribute to the development of future physical diseases [39],[40].

The high MRR of unnatural death from external causes, in particular suicide, was seen in individuals who had lost a parent owing to suicide, which may reflect a heritability of mental health problems or familial transmission of impulsive aggression [14],[15],[18]. In addition, social-behavioral consequences of parental death, such as the loss of a care giver, misbehaviors, and functioning impairment [20],[41]–[45] can increase the risk of death from injuries or other external causes. For example, parental suicide was associated with more transport accident deaths in our population. Furthermore, psychosocial stressors embedded with parental death in childhood [20],[41]–[44] can interact with genetic susceptibility to create psychiatric problems [46],[47], which may lead to more deaths from external causes. Unnatural parental death impacted child's mortality more than natural parental death, which may be explained by the fact that unexpected or sudden parental death has more severe psychosocial consequences [15],[20],[41],[48].

Reactions to bereavement are expected to diminish but not always to disappear over time [19],[20],[49]. It is not clear how long parental death in childhood will affect offspring's mortality and previous research often focused on offspring's short-term reactions and survival [6]–[8]. Our study showed not only short-term risks but also elevated risks until early adulthood or even mid-adulthood. Detrimental effects on mortality from physical diseases manifested more obviously in the oldest bereaved group in a later stage of the follow-up period, probably because of the fact that most diseases develop at an advanced age. We also observed that short-term mortality from external causes varied with the child's age at bereavement, with the youngest age group suffering most, which indicates lack of intense care immediately after bereavement for very young children. The above observations highlight the fact that children's coping mechanisms and their understanding of death are related to their age-dependent developmental capacity [41],[49]. Yet, the overall risk of death remained high for more than 20 years independently of age at bereavement.

Our study has a number of strengths. We combined nationwide data from three Nordic countries on virtually all study participants who were followed for up to 42 years without loss to follow-up [27],[50]. Information on exposure and outcome is virtually complete and of very high quality in the Nordic countries, which makes our estimates on long-term mortality accurate [27],[50]. The detailed information on cause of death permitted us to examine the type of death as well as major groups of cause of death. Furthermore, we were able to take a number of important factors into account, such as socio-demographic characteristics of parent and child, as well as other potential confounders.

Our findings should also be interpreted in the light of limitations. First, we lacked information on some baseline factors, such as the quality of the parent-child relationship, common lifestyle factors, and the physical environment like residential settings. We hope that adjustment for maternal factors (education and social status) and family size (parity) may reduce the effects of these unmeasured confounders to some extent. Second, we had no data on post-loss changes in families, network, lifestyle factors, or risky behaviors, etc. However, these changes may be on the pathways from exposure to outcome, which should not be adjusted [51]. Third, this study only presents the associations between parental death and child death for overall mortality and for major groups of causes of death. The single disease-specific associations would vary to different extents according to combinations of age, sex, and the length of follow-up period, and such analyses would need much more space to present, which are beyond the scope of this study. Last, we do not have data to identify the subgroups who were able to manage this life event well, for evaluating the positive outcomes of parental death in childhood as an indication of resilience [19]. However, our aim is to present average estimates for the association at the entire population level.

To conclude, parental death in childhood was associated with a long-lasting increased mortality risk from both external causes and diseases, regardless of age and sex of the child and the deceased parent, cause of parental death, as well as population characteristics like socioeconomic background. It should be acknowledged that that the increased mortality represents only the tip of the iceberg effects [3], therefore even a small increased risk of mortality may indicate substantial adverse impacts on personal behaviors, health and social well-being, and family situations in the bereaved populations. The findings warrant the need for health and social support to the bereaved children and such support may need to cover an extended time period. This information should be taken into account for medical and public health professionals when considering clinical responses and public health strategies.

## Abbreviations

ICD: International Statistical Classification of Diseases and Related Health Problems (ICD) code
MRR: mortality rate ratio
